# MANAGEMENT OF DIFFICULT URTICARIA

**DOI:** 10.4103/0019-5154.55641

**Published:** 2009

**Authors:** Sudha Yadav, A K Bajaj

**Affiliations:** *From the Departments of Skin, Veneral Disease, and Leprosy, M.L.N. Medical College, Allahabad, UP, India.*

**Keywords:** *Chronic idiopathic urticaria*, *difficult urticaria*, *urticaria*

## Abstract

Chronic urticaria, a major health problem causing patient's distress, induces often physicians' dilemma while dealing with its etiology, investigations and management. Clinical approach of such cases should include apart from clinical history and physical examination laboratory investigations like routine blood test, thyroid profile, etc. as well as sometimes special test like autologous serum skin test. Management includes reassurance, avoidance of precipitating factors, treatment of underlying disorders, and non-pharmacological approach along with pharmacotherapy. First line drug therapy comprises non-sedative and sedative antihistamines, second line doxepin, nifedipine, leukotriene-inhibitors, sulfasalazine, etc. and third line cyclosporine, dapsone, colchicin, etc.

## Introduction

Ordinary chronic urticaria is appropriately termed as a “Cinderella” disease with a capricious course and a demoralizing response to treatments. There are no guaranteed means of controlling attacks. It can be difficult to determine the appropriate medication, as the condition is intermittent and outbreaks also typically clear up without any treatment.

There are several recognizable clinical patterns of urticaria. It is characterized by the rapidity of its fluctuation. Urticaria becomes chronic when wheals fluctuate daily or almost daily for six weeks or more. It may occur continuously for a long duration or daily for a few days and then not for a week or two, just to reappear again. When continuous urticarial activity is punctuated by gaps of several weeks or months or, in other words, if the episodes are of shorter duration than the symptom-free period, it is better to name it episodic or recurrent. Recognizing this pattern may be helpful in identifying an external trigger factor.

Chronic urticaria remains a major problem in terms of etiology, investigation, and management. It may cause considerable distress and may last for years, but it can often be alleviated by appropriate management. The remainder of this article will review the management of chronic urticaria excluding autoimmune urticaria, contact urticaria, and delayed pressure urticaria, as these topics are discussed elsewhere.

## Investigations

We still do not know the etiology in a majority of urticaria patients. Establishing the cause of chronic urticaria is difficult and at times almost impossible. A “Practice Parameter” for the diagnosis and management of chronic urticaria was published in 2000, emphasizing the conditions that need to be considered in the differential diagnosis.[1] The most important part in the investigation of urticaria is a detailed and comprehensive history and thorough physical examination. The history itself can be regarded as the most valuable diagnostic tool in identifying the cause of chronic urticaria.[2] It should include information regarding the duration of disease, frequency of attacks, duration and distribution of individual lesions, food and food additives, occupation and leisure activities, previous treatment, known adverse reactions, enquiry about the potential source of infection, associated illness, for example, fever, joint pain and so on, past and family history of an atopy, thyroid, and other autoimmune diseases, and an assessment of the impact of the disease on the patient's quality of life. A full physical examination looking for the morphology and duration of wheals, bruising, livedo reticularis, and signs of systemic disease should always be undertaken.[3]

Routine laboratory screening does not contribute substantially to the diagnosis and etiology of chronic urticaria.[4] It has been shown that extensive investigations are not often required and are not cost effective also. Investigation of chronic urticaria should be guided by the clinical presentation. Most patients with antihistamine responsive chronic urticaria do not need a plethora of extensive investigations. Only a complete blood count and differential white cell count and erythrocyte sedimentation rate (ESR) should be performed routinely. Eosinophilia would prompt a search for a parasitic disease, and an elevated ESR suggests the possibility of an underlying systemic disease (may be raised in urticarial vasculitis and definitely raised in systemic lupus erythematosus). Thyroid autoantibodies should be considered and thyroid function tests must be performed for all patients with chronic urticaria not responding to antihistamines, especially women and patients with a family history of thyroid disease or other autoimmune disorders.[5]

Autologous serum skin test (ASST) should be performed in all antihistamine-nonresponders. The ASST has a sensitivity of 70% and a specificity of 80%. A positive test is suggestive, but not diagnostic of an autoimmune basis for the patients' chronic urticaria.[6] Skin biopsy is not necessary for routine management of clinically typical urticaria that responds to antihistamines, but it should be performed in all patients with lesions lasting for more than 24 hours, to confirm the diagnosis of urticarial vasculitis.

## Therapeutic Modalities

Chronic idiopathic urticaria is a frequent problem where response to treatment is often disappointing. Treatment of patients with chronic idiopathic urticaria involves reducing symptoms with least invasive therapy and carefully balancing risk and benefit. The goal of the therapy is to reduce symptoms until spontaneous resolution occurs. Management of a patient with chronic idiopathic urticaria can be both frustrating and rewarding. The initial management of chronic urticaria should be directed according to the results of a full clinical assessment. A step by step approach should be taken.

Many pharmacological and nonpharmacological interventions are available, but none is accepted universally. For this reason, the treatment regime should be tailored to individual patients. The key features of all regimes should be removal of any identifiable cause, avoidance of aggravating factors, advice, explanation, information, and reassurance.

### Advice and information

Treatment of chronic urticaria presents a challenge to both physicians as well as patients. Advice on general measures and information can be helpful to most patients with chronic urticaria. Reassurance is another important aspect of therapy, as appearance of urticarial rash is often more frightening than the generally favorable prognosis warrants. Also some patients become quite distraught because of their own inability to figure out what is bringing on their hives and many get more discouraged by the physician's apparent inability to delineate a specific cause.

Patients should be informed that: (1) although the diagnosis is clear, in most cases the cause may not be identifiable; (2) the condition is not contagious, serious, or a sign of cancer; (3) unless it is related to underlying systemic disease, the condition will resolve eventually. A clear explanation that chronic ordinary urticaria is not an allergic problem is often a useful point to address and failure to do this often leads to dissatisfaction, with the fact that allergy tests are not offered immediately.

### Avoidance of aggravating factors

Most treatment plans for urticaria involve being aware of one's own triggers, but this can be difficult or impossible. Obviously, the preferred form of treatment is avoidance of the causative agent in the exceptional case where it can be identified and avoidance is feasible. In addition to avoidance of the possible causative factor, patients with chronic urticaria should be appropriately advised to avoid, to the extent feasible, common potentiating factors, such as, alcohol overuse, excessive tiredness, emotional stress, overheated surroundings, aspirin, and other nonsteroidal anti-inflammatory drugs (NSAIDs).

Avoidance of aspirin and other NSAIDs should usually be recommended, because these drugs aggravate chronic urticaria in 30% of the patients.[7–8] Regular low-dose aspirin, for its antithrombotic properties, can be continued. Angiotensin converting enzyme inhibitors should be avoided in chronic urticaria because angioedema and, rarely, urticaria are recognized adverse effects.[9]

Avoidance of dietary pseudoallergens including food additives (azo dyes, benzoate preservatives), natural salicylates (present especially in fruits, beer, and wine), and unidentified aromatic substances in tomatoes, herbs, and white wine have not been found beneficial in treating chronic urticaria.[10–11] A strict low pseudoallergen diet for 3 weeks initially may be helpful for some patients with mild chronic urticaria, but there is a general agreement that dietary measures have no role in most forms of chronic urticaria unless proved by a double blind placebo-controlled challenge.

### Treatment of underlying diseases

Chronic urticaria may be a manifestation of an underlying disease, and in these cases, treatment of the underlying condition may be warranted. The best example of a systemic condition that is commonly associated with chronic urticaria is autoimmune thyroid disease, that is, Hashimoto's thyroiditis. Treatment with thyroxine has been reported to alleviate the urticaria.[12] A few reports have suggested that *Helicobacter pylori* might be associated with chronic urticaria in some patients, and eradication of *H. pylori* is associated with remission of chronic urticaria.[13–14] According to one report, five patients with chronic urticaria went into complete remission with oral acyclovir therapy.[15] Use of nasal filters in treating chronic urticaria has occasionally been found beneficial.[16] A few case reports have described resolution of recalcitrant chronic urticaria after treatment of dental abscesses.[17]

## Nonpharmacological Approaches

Similar too many other therapeutically challenging disorders, chronic idiopathic urticaria has seen an abundance of fad therapies, including ayurvedic and homeopathic medications and naturopathy come and go.

Frequent tepid showers and application of soothing lotions can be prescribed as cooling agents when wheals erupt and are most pruritic.[18] These include 0.5-1% menthol or calamine in aqueous cream/lotion and 10% crotamiton lotion. Use of antihistamine creams is not found to be effective. According to few studies, the use of a topical steroid may help in reducing wheal response.[19] Phototherapy with ultraviolet light or photo chemotherapy (PUVA) has been used for treating chronic urticaria, but the reported results have been inconclusive.[20–21] A complementary psychological treatment of patients suffering from chronic idiopathic urticaria seems necessary, because of the high prevalence of psychological symptoms. Relaxation under hypnosis has produced a decrease in itching, but not in the number of hives.[22]

## Pharmacotherapy

A practical approach to chronic idiopathic urticaria bases its treatment on the severity of the disease and the patient's previous response to therapy. The mainstay of this therapy is the use of antihistamines that are considered as first-line drugs for treating chronic idiopathic urticaria. Second-line drugs include systemic corticosteroids, leukotriene antagonists, mast cell stabilizer, doxepin, thyroxine, nifedipine, terbutaline, sulfasalazine, and so on.[23–24]

## First-line antihistamine therapy

Histamine is the main mediator of urticaria and antihistamines are reasonably effective for treating patients with chronic urticaria. Various studies on the role of antihistamines in chronic urticaria showed a 44 to 90% response rate.[12] To get consistent results, an antihistamine should be taken on a daily basis and not as and when required, the frequency depending upon their half-life. In other words, they should not be taken only when the patient is symptomatic. Antihistamines can be grouped into:-

- Classic (sedating) H-1 antihistamine—chlorpheniramine, hydroxyzine, diphenhydramine.
- Second/Newer generation (nonsedating) H-1 antihistamine—loratadine, cetirizine, terfenadine, mizolastine.
- Second generation H-1 antihistamine derivatives—desloratadine, levocetirizine, fexofenadine.
- H-2 antihistamines—cimetidine, ranitidine.

The newer generation H-1 antihistamines with less sedating and less anticholinergic effects are preferred over the older generation H-1 antihistamines, as the initial choice of therapy. Combining drugs from the different groups and with nonhistaminic drugs can lead to a better control of urticaria. Treatment should be started with a nonsedating antihistamine at the licensed dosage, taken at the same time each day. Some of the workers prefer to combine newer antihistamine with classic sedating antihistamine at night and also with H-2 antihistamine. Addition of H-2 antihistamine may sometimes provide a better control of urticaria than an H-1 antihistamine taken alone. Although in practice, it may be more helpful for the dyspepsia that may accompany severe urticaria. H-2 antihistamines alone have no effect on histamine-induced pruritus, hence should not be used on their own.[3] It has become common practice to double or even triple the dosage of nonsedating antihistamine for patients who respond poorly, on the grounds that their additional anti-allergic and anti-inflammatory properties may be clinically beneficial at these higher doses.[25]

It is best to avoid all antihistamines in pregnancy, especially during the first trimester, although none has been shown to be teratogenic in humans. Hydroxyzine is the only antihistamine to be specifically contraindicated during the early stages of pregnancy. Chlorpheniramine is often chosen for pregnant women when antihistamines are essential, because it has a long safety record. None of the currently licensed anti-histamines is contraindicated in children 12 years and older. Short acting classic antihistamines still have a place in the primary management of urticaria in children between the ages of 1 and 6 years.[26–27]

Cetirizine and levocetirizine should be avoided in severe renal impairment (creatinine clearance <10ml/min), whereas, loratadine and desloratadine should be used with caution. Mizolastine is contraindicated by significant hepatic impairment. Clinically important drug interactions are infrequent, but coadministration of mizolastine with systemic erythromycin and ketoconazole is contraindicated, because of a concern about the possible occurrence of cardiac arrhythmia, as noted with terfenadine and astemizole. Caution is also advised with use of other hepatic cytochrome P450 3A4 inhibitors or substrates, including cimetidine, cyclosporine, and nifedipine, all of which may be used for urticaria in special situations.[3]

### Second-line therapy

Sixty percent of the patients with chronic urticaria respond well or reasonably well to antihistamines, but 40% derive little or no relief. There are many possible second-line drugs to consider when urticaria does not respond to antihistamines. Helping these patients is challenging and is influenced considerably by a full clinical assessment, experience, and thorough knowledge of the literature of the clinician. It is unusual for children to require second-line drugs, except for the occasional use of systemic corticosteroids for very severe, acute exacerbations of chronic urticaria. Second-line drugs should be used with considerable care in pregnancy.[3]

Doxepin is a tricyclic antidepressant with very potent H-1 and H-2 antihistaminic properties. Doxepin is more effective than diphenhydramine at a dosage of 10 mg thrice daily. Doxepin should not be advised after a recent myocardial infarction or for patients with severe liver disease. It is best to start at a low dose of 10 mg daily at night, which can be increased to 20 or 30 mg a day.[28–29] Mirtazepine is an antidepressant that demonstrates a significant effect on the H-1 receptor and has antipruritic activity as well.[30]

β-agonists like terbutaline and epinephrine also have been combined with H-1 antihistamine in a small number of patients,[31] although there is no mention in the recent literature of their use in chronic urticaria.

Nifedipine is a calcium channel blocking drug that possesses mast cell stabilizing properties as well. Nifedipine should be started at a dosage of 5 mg thrice daily and can be increased up to 20 mg thrice daily.[32]

The use of prednisolone in the treatment of urticaria is a controversial issue. It is effective for nearly all presentations of chronic urticaria, but often needs to be used at high doses to achieve a good control of urticaria. A short course of systemic steroids (30-40 mg of prednisolone/day) can be given in resistant cases of chronic urticaria that have not responded to antihistamines. Patients must understand that although systemic steroids may be the only treatment that completely eliminates their lesions, long-term use is unacceptable because of their known adverse effects, such as, diabetes mellitus, hypertension, osteoporosis, and gastrointestinal bleeding.[33–34]

Leukotriene receptor antagonists zafirlukast and montelukast have been seen to have a beneficial effect in the treatment of a few cases of chronic urticaria. The daily dosage of montelukast is 10 mg once a day at bed time and that of zafirlukast is 20 mg twice a day. However, most studies do not support their role in treating chronic urticaria.[35–37] Zileuton, a 5-lipooxygenase inhibitor that inhibits leukotriene generation, has been found to be effective in improving chronic urticaria.

Sulfasalazine has been reported for the treatment of steroid-dependent chronic urticaria, ordinary urticaria. The usual starting dosage is 1 gm twice daily, leading to a maximum regular dosage of 4 gm daily.[38]

Thyroxine supplementation has been reported to suppress chronic ordinary urticaria in biochemically euthyroid patients with antithyroid autoantibody. The dosage of thyroxine used in the studies varied from 50 mg to 250 mg daily. Thyroid function tests should be done before starting the treatment and rechecked after four to six weeks of therapy.[5]

Rofecoxib, a newer cyclo-oxygenase-2 (COX-2) inhibitor, has shown promise in treating patients with refractory chronic idiopathic urticaria.[39]

### Third-line therapy

Third-line therapy typically involves the use of immunomodulatory drugs for treating refractory chronic urticaria.[33] These agents include cyclosporine, cyclophosphamide, methotrexate, mycophenolate mofetil, oral tacrolimus, intravenous immunoglobulin (IVIg), dapsone, colchicine, plasmapheresis, hydroxychloroquine, warfarin, omalizumab, and so on.

Cyclosporine has been shown to be effective in severe unremitting urticaria that has had a poor response to conventional treatment with antihistamines. Cyclosporine 3-5mg/kg/day appears to benefit about two-thirds of patients with chronic urticaria who do not respond to antihistamine.[40]

High dose of intravenous immunoglobulin (IVIg) and plasmapheresis have been found to be associated with some apparent benefits in the treatment of chronic urticaria.[41–42]

Dapsone, colchicine, hydroxychloroquine, oral tacrolimus, cyclophosphamide, and low dose methotrexate, which have immunomodulatory properties, are effective in the treatment of chronic urticaria.[24,33,43–45]

A recent report by Shahar *et al.,* documented a significant improvement in nine patients with chronic urticaria after they had taken mycophenolate mofetil for 12 weeks.[46]

A few studies have found that warfarin appears to have beneficial effects in some patients with chronic urticaria.[47]

Omalizumab, a humanized monoclonal antibody, has recently been reported to have been effective in three patients with chronic urticaria.[48]

In a recent placebo controlled study, autohemotherapy has shown a beneficial role in ASST positive chronic urticaria patients.[49] Autologous serum therapy (AST), a modified form of autologous whole blood therapy, has also been found to be fairly effective in chronic urticaria.[50]
